# A novel role of kynureninase in the growth control of breast cancer cells and its relationships with breast cancer

**DOI:** 10.1111/jcmm.14547

**Published:** 2019-07-22

**Authors:** Yingzhe Liu, Xueping Feng, Jinping Lai, Wenjun Yi, Jiu Yang, Tao Du, Xueying Long, Ye Zhang, Yongzhi Xiao

**Affiliations:** ^1^ Xiangya International Medical Center, National Clinical Research Center for Geriatric Disorders, Xiangya Hospital Central South University Changsha China; ^2^ Department of Oncology and Institute of Medical Sciences, National Clinical Research Center for Geriatric Disorders, Xiangya Hospital Central South University Changsha China; ^3^ Department of Pathology, Immunology, and Laboratory Medicine University of Florida Gainesville Florida; ^4^ Department of General Surgery, The Second Xiangya Hospital Central South University Changsha China; ^5^ Department of Ultrasonography, The Second Xiangya Hospital Central South University Changsha China

**Keywords:** breast cancer, KYNU, tumour suppression

## Abstract

Breast cancer is the most common malignancy among women worldwide. Kynureninase (KYNU) located in 2q22.2, which was associated with tryptophan utilization and metabolic diseases including cardiac, renal and limb defects syndrome 2. However, the role of KYNU in breast cancer (BC) development remains unclear. The expression of KYNU was examined by immunohistochemistry (IHC) in 137 primary BC tissues, and the correlation of KYNU expression with clinical pathological characteristics and the biomarkers (ER, PR, HER2, E‐cad and Ki‐67) was analysed. The role of KYNU in cancer cell proliferation, tumour growth and development was evaluated by MTT assay, soft agar colony formation assay and xenograft mouse models. Among 137 primary BC tissues, 46.7% (64/137) had high KYNU expression (IHC scores >4) while 53.3% (73/137) had low KYNU expression (IHC scores ≤4). The expression of KYNU was positively correlated with the expressions of ER (*P* = .002), PR (*P* = .007) and E‐cad (*P* = .03), while negatively associated with tumour grade (*P* = .008), tumour stage (*P* < .001) and the expressions of HER2 (*P* = .04) and Ki‐67 (*P* = .019). Overexpression of KYNU significantly inhibited cell proliferation in cell culture, colony formation in soft agar and xenograft BC development in NOD/SCID mice. Kynureninase suppresses BC cell proliferation, tumour growth and development. Kynureninase may function as a tumour suppressor in BC.

## 1 INTRODUCTION

Breast cancer (BC) is the most common diagnosed cancer and the fifth leading cause of cancer death among women in China.^1^ Although the rapid development of target therapy and immunotherapy in recent years, surgery, hormone therapy, radiotherapy and chemotherapy are still the effective options for the treatment of BC.^2^ The 5‐year survival rate of early‐stage breast cancer (EBC) patients in China is about 58%‐78%.^3^

Breast cancer is a clinically heterogeneous disease. However, BC cells from the cancerous lump may have spread to other organs of patients after different kinds of treatment (a mastectomy, radiotherapy and chemotherapy). Although tumour size, histopathological grades and clinical stages are important indicators for clinical management of BC, it is a common phenomenon that histologically similar tumours may have different prognoses and respond to therapy differently, due to the molecular difference among the histologically similar tumours.

Kynureninase (KYNU) or L‐kynurenine hydrolase belongs to the kynureninase family. Kynureninase gene is located at 2q22.2.^4^ KYNU is a pyridoxal‐5′‐phosphate (pyridoxal‐P)‐dependent enzyme that catalyses the cleavage of L‐kynurenine and L‐3‐hydroxykynurenine into anthranilic and 3‐hydroxyanthranilic acids, respectively. Kynureninase is involved in the biosynthesis of NAD cofactors from tryptophan through the kynurenine pathway.^5^

Kynureninase was associated with tryptophan utilization and metabolic diseases. It has been reported that KYNU is associated with metabolic neurological,^6^ cardiac^7^ and renal disease.^8^ Inhibition of the kynurenine pathway protects against reactive microglial‐associated reductions in the complexity of primary cortical neurons.^9^ KYNU has been reported to be down‐regulated in the highly aggressive osteosarcoma cell lines.^10^ The association of KYNU with cancer is rarely reported, and the role of KYNU in cancer development is unclear.

Here, we report that the expression of KYNU is negatively correlated with clinical BC histological grades and tumour stages. Overexpression of KYNU inhibits BC cell proliferation, colony formation and xenograft tumour development in mouse models. Kynureninase may function as a tumour suppressor in BC.

## 2 MATERIALS AND METHODS

### 2.1 Breast cancer tissue samples

Formalin‐fixed paraffin‐embedded (FFPE) BC samples with detailed clinical and pathological data from 137 BC patients treated at the second Xiangya Hospital, Central South University, during April 2012‐March 2017, were collected. This study has been approved by the Research Ethics committee of the second Xiangya Hospital and the Xiangya Hospital, Central South University.

### 2.2 Immunohistochemistry (IHC) analysis

For IHC, briefly, 4‐μm‐thick paraffin‐embedded tissue sections were treated with an antigen retrieval solution (eBioscience, #00‐4955‐58) and stained by using a Vectastain^®^ abc kit, according to the manufacturer's protocol (Vector Laboratories, # PK‐4000). The tissue sections were incubated with the primary antibodies (Table 1) in 10% blocking serum overnight at 4°C and then incubated with biotinylated secondary antibody (1:500 dilution) 60 minutes, followed by avidin‐biotin peroxidase complex (DAKO) 30 minutes. Finally, tissue sections were stained with 3,3′‐diaminobenzidine (DAB, Sigma, #D12384) and counterstained with Harris' modified haematoxylin.

**Table 1:** Antibodies and staining conditions in the study

Antibody	Company	Clone	Dilution	Antigen retrieval	Incubation
KYNU	GeneTex, Inc (North America; Cat No. GTX33291)	Rabbit Polyclonal	1:1000	Standard	overnight at 4°C
ER	Ventana Medical Systems (Tucson, USA)	Rabbit Monoclonal SP1	Pre‐dilution	Standard	37°C, 60 min
PR	Ventana Medical Systems	Rabbit Monoclonal 1E2	Pre‐dilution	Standard	37°C, 60 min
HER‐2	Ventana Medical Systems	Rabbit Monoclonal 4B5	Pre‐dilution	Standard	37°C , 60 min
E‐cad(E‐Cadherin)	MXB Biotechnology MAB‐0589	Mouse Monoclonal 4A2C7	Pre‐dilution	Standard	37°C , 60 min
Ki‐67	Ventana Medical Systems	Rabbit Monoclonal 30‐9	Pre‐dilution	Standard	37°C , 60 min

ER, PR and HER2 staining were scored according to the American Society of Clinical Oncology (ASCO) and the College of American Pathologists (CAP) guidelines. Samples with at least 1% positive cells for ER or PR staining were classified as ER or PR positive.^11^ HER2 staining was scored^12^ as 1 negative, 2 as equivocal, 3 as positive; for Ki67, if ≥15% of the nuclei were stained, sample was classified as Ki67‐positive (high) expression.^13^ E‐cad expression was considered positive if positive cells is equal to or more than 50% continuous membrane staining was present in the breast cancer cells, and negative or low expression if less than 50%, which was the median percentage observed in the included subjects.^13^ The KYNU protein was found to be expressed primarily in the cytoplasm of tumour cells. The cytoplasm staining fraction (CF) was assigned a score of 0 (0%–5%), 1 (5%–25%), 2 (26%–50%), 3 (51%–75%) or 4 (>75%), and cytoplasm staining intensity (CI) was noted as 0 (negative), 1 (weak), 2 (moderate) and 3 (strong). Subsequently, a combined Cytoplasm Score (CS) was calculated by multiplying CF and CI (range of 0‐12). For statistical analyses, the cut‐off values for KYNU expression were chosen on the basis of heterogeneity using the log‐rank test for OS. The optimal cut‐off value was determined as low (scores ≤4) or high (scores >4) KYNU expression.

### 2.3 Cells and reagents

Breast cancer MCF‐7 and MDA‐MB‐231 cell lines were purchased from American Type Culture Collection (ATCC). They were cultured in DMEM high glucose medium supplemented with 1% HEPES, 1% Pen/Strep, 1% sodium pyruvate and 10% foetal bovine serum (FBS) and incubated at 37°C in a humidified atmosphere containing 5% CO2. MCF‐7 and MDA‐MB‐231 cells stably expressing control vector (pcDNA3.1), pcDNA3.1‐KYNU, were cultured in DMEM supplemented with high glucose medium supplemented with 1% HEPES, 1% Pen/strep, 1% sodium pyruvate, 10% FBS and G418 (1000 g/mL).

### 2.4 Transfections

The transfection of pcDNA‐KYNU (OriGene #RC214932) was performed by using the TransIT‐LT1 transfection reagent (Mirus) according to manufacturer's recommendations. Stable transfection was achieved by G418 selection. For siRNA experiment, siRNAs was purchased from Santa Cruz (#sc‐51370).

### 2.5 Western blot analysis

Cell lysates were separated by SDS‐PAGE and transferred to PVDF membranes. Immunostaining was done using antibodies specific for KYNU (GeneTex, Lot No. 33291). Chemiluminescence detection was performed by using the Pierce ECL Western blotting substrate (Thermo Scientific).

### 2.6 Cell proliferation assay

Cell proliferation was measured by using CellTiter 96^®^ Non‐Radioactive Cell Proliferation Assay kit (Promega) according to manufacturer's instructions. Briefly, after the cells were cultured to different time points, the dye solution was added into the 96‐well plates. The plates containing dye solution were incubated at 37°C cell culture incubator for 4 hours. The solubilization solution/stop mix was added to the culture plates, and the plates were measured at 570 nm with reference wavelength of 630 nm using a 96‐well plate reader.

### 2.7 Soft agar colony formation assay

Cells (1 × 10^3^) were seeded in medium containing 0.4% agarose (GenePure LowMelt Agarose, ISC BioExpress) on top of 0.8% agarose/media. After solidifying, the cells were covered with media. Media was changed every 3 days. Three weeks later, colonies were stained with 0.01% crystal violet (Alfa Aesar). Colonies larger than 50 μm were enumerated.

### 2.8 Breast cancer xenograft mouse models

NOD‐SCID female mice (6‐week‐old) were used for xenograft model. To examine the role of KYNU development, female mice were subcutaneously inoculated with 5 × 10^6^ MDA‐MB‐231 cells stably expressing control vector (Control) or KYNU overexpression (KYNU‐OE). Five mice for each group were included. Tumour volume was estimated by the formula volume = (*l* × *w* ^2^)/2, where *l* stands for length and *w* stands for width. Tumour growth was monitored and measured every 3 days.

### 2.9 Statistical analyses

Statistical analyses were performed using SPSS version 19.0 for Windows (IBM Corp.). The correlations between KYNU expression and clinicopathological characteristics were evaluated using the chi‐square test. The association of KYNU expression with tumour grade and TNM stage of breast cancer was calculated using one‐way analysis of variance. *t* test was used to analyse MTT assay and the growth in xenograft mouse model for cell proliferation.

## 3 RESULTS

### 3.1 The differential expression of KYNU in breast cancer and benign cells

**Figure 1:**
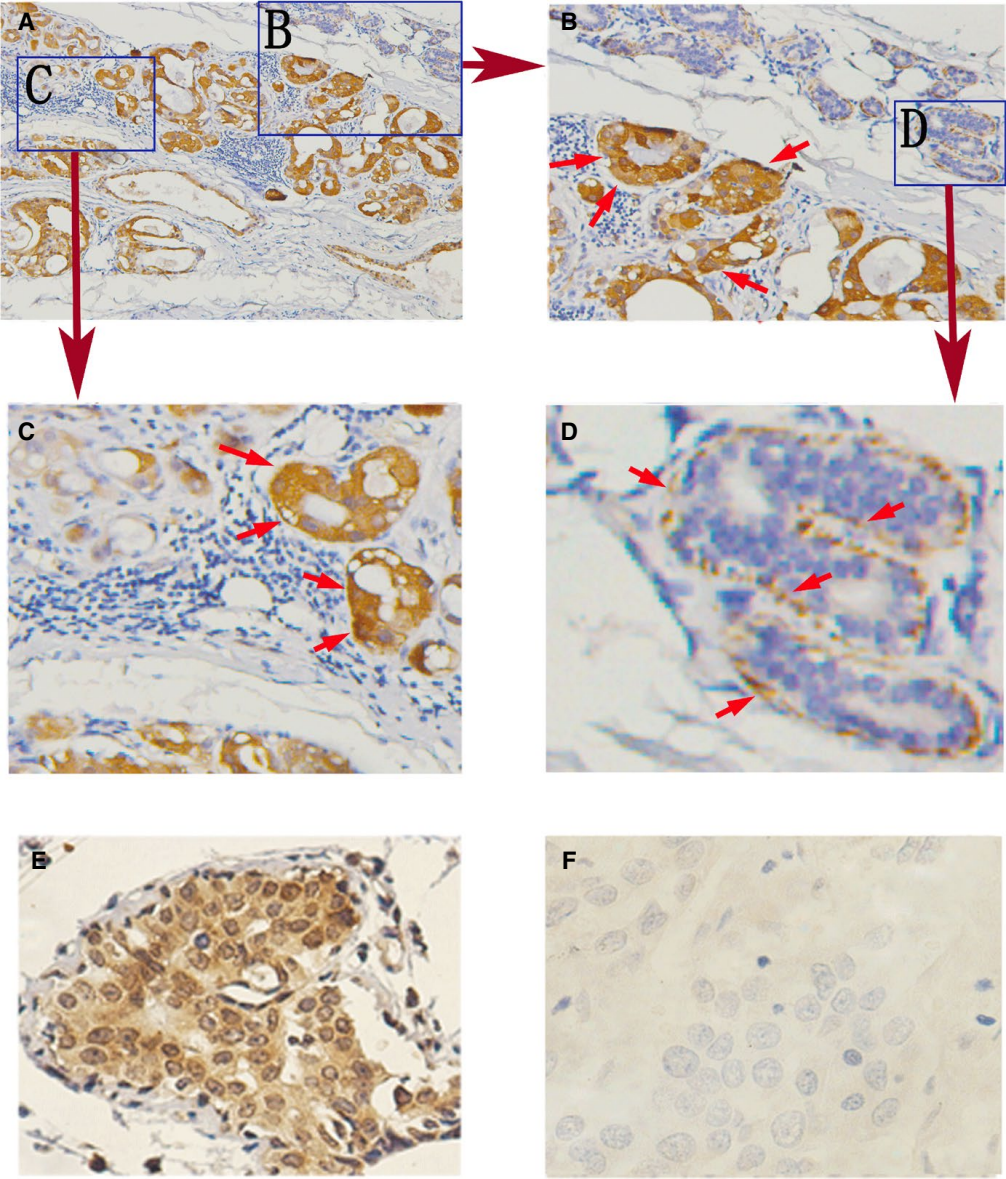
The expression of KYNU in breast cancer cells and benign myoepithelium cells: (A‐E) High: KYNU protein expression in mammary ductal carcinoma cells (A, B, C, E), but not in lymphocyte and benign mammary gland cells (C, D). (E, F) KYNU expression in differentiated BC cells, high KYNU expression in well‐differentiated BC cells (E), but not in poor‐differentiated BC cells (F)

Among all sections analysed, we found that KYNU protein was highly expressed both in mammary ductal carcinoma cells (Figure 1A,C) and in benign myoepithelium cells (Figure 1D), but not in lymphocyte and benign mammary gland luminar cells (Figure 1C,B,D).

The expression of KYNU was very high in well‐differentiated BC cells (Figure 1E) and very low in poor‐different BC cells (Figure 1F).

### 3.2 The expression of KYNU is positively correlated with BC differentiation but negatively with BC tumour grade and tumour stage

**Figure 2:**
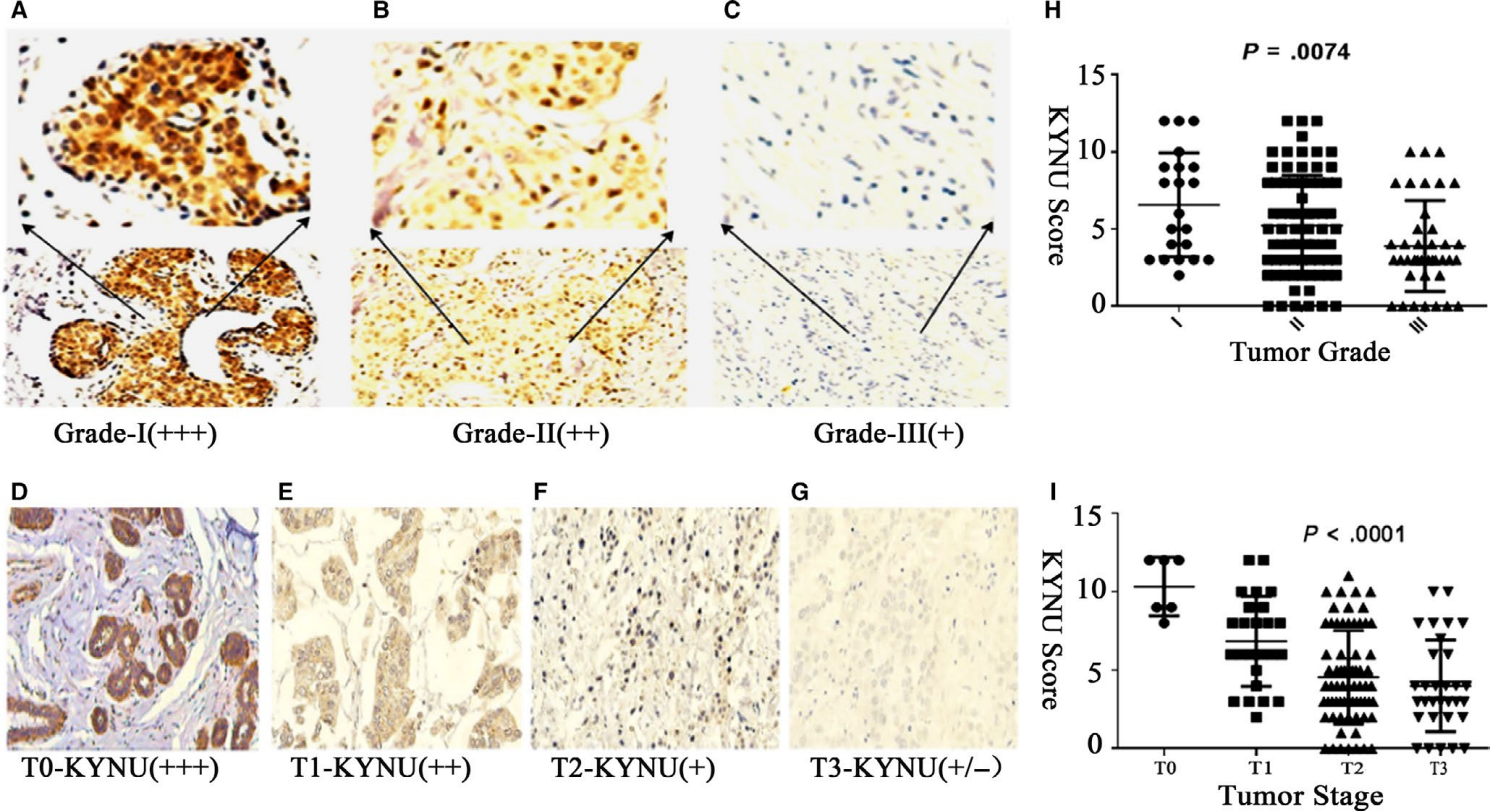
KYNU is differentially expressed among BC tumour grades and among BC tumour stages. A‐C, The expression of KYNU in well‐differentiated BC (Grade I) (A), the middle‐differentiated (Grade II) (B) and poor‐differentiated (Grade III) (C) BC. D‐G, The expression of KYNU in stage T0 (D), T1 (E), T2 (F) and T3 (G) BC. (H) The analysis of the correlation between KYNU expression and tumour grades. I, The analysis of the correlation between KYNU expression and tumour stages. Microscopic image was at 200× magnitude; one‐way analysis of variance

As shown in Figure 2, KYNU was highly expressed in well‐differentiated BC (Grade I) (Figure 2A) while its expression was medium in middle‐differentiated BC (Grade II) (Figure 2B) and very low or lost in poor‐differentiated BC (Grade III) (Figure 2C). The expression of KYNU was negatively correlated with the histopathological grades of BC (Figure 2H). Similarly, the expression of KYNU was very high in T0 BC (Figure 2D), high in T1 BC (Figure 2E), while its expression was low in T2 BC (Figure 2F) and very low in T3 BC (Figure 2G). The expression of KYNU was reversely related to tumour stage (T stage) (Figure 2I).

### 3.3 The association of KYNU expression with breast cancer clinicopathological characteristics

Typical clinicopathological parameters and immunohistochemical staining of low or high KYNU expression are shown in Table 2. Among 137 primary BC tissues, 46.7% (64/137) had high KYNU expression (IHC scores >4), while 53.3% (73/137) showed low KYNU expression (IHC scores ≤4). As summarized in Table 2, the expression of KYNU was positively associated with the expression of ER (*P* = .002), PR (*P* = .007) and E‐cad (*P* = .03), while it was negatively related to the expressions of HER2 (*P* = .04) and Ki‐67 (*P* = .019). However, the expression of KYNU was not significantly related to patients' age (year) or N‐stage(N) of BC.

**Table 2:** Other Correlation of KYNU expression with BC clinicopathologic characteristics

Characteristic	KYNU expression		*P*‐value
	Low expression No. (%)	High expression No. (%)	
Age (y)*	51 (36‐73)	49 (35‐84)	.56
N‐stage			
N0	23/111 (20.7%)	22/111 (19.8%)	.422
N1	21/111 (18.9%)	22/111 (19.8%)	
N2	10/111 (9.0%)	7/111 (6.3%)	
N3	5/111 (4.5%)	1/111 (0.90%)	
Oestrogen receptor			
Positive	34/134 (25.4%)	54/134 (40.3%)	.002
Negative	31/134 (23.1%)	15/134 (11.2%)	
Progesterone receptor			
Positive	28/126 (22.2%)	41/126 (32.5%)	.007
Negative	37/126 (29.4%)	20/126 (15.9%)	
HER‐2			
Positive	40/99 (40.4%)	30/99 (30.3%)	.04
Negative	10/99 (10.1%)	19/99 (19.2%)	
E‐cad			
≥50%	9/78 (11.6%)	16/78 (20.5%)	.03
<50%	33/78 (42.3%)	20/78 (25.6%)	
Ki‐67			
≥15%	38/97 (39.2%)	25/97 (25.8%)	.019
<15%	12/97 (12.4%)	22/97 (22.7%)	

* Median (range).

### 3.4 The association of KYNU expression with other biomarkers of BC

**Figure 3:**
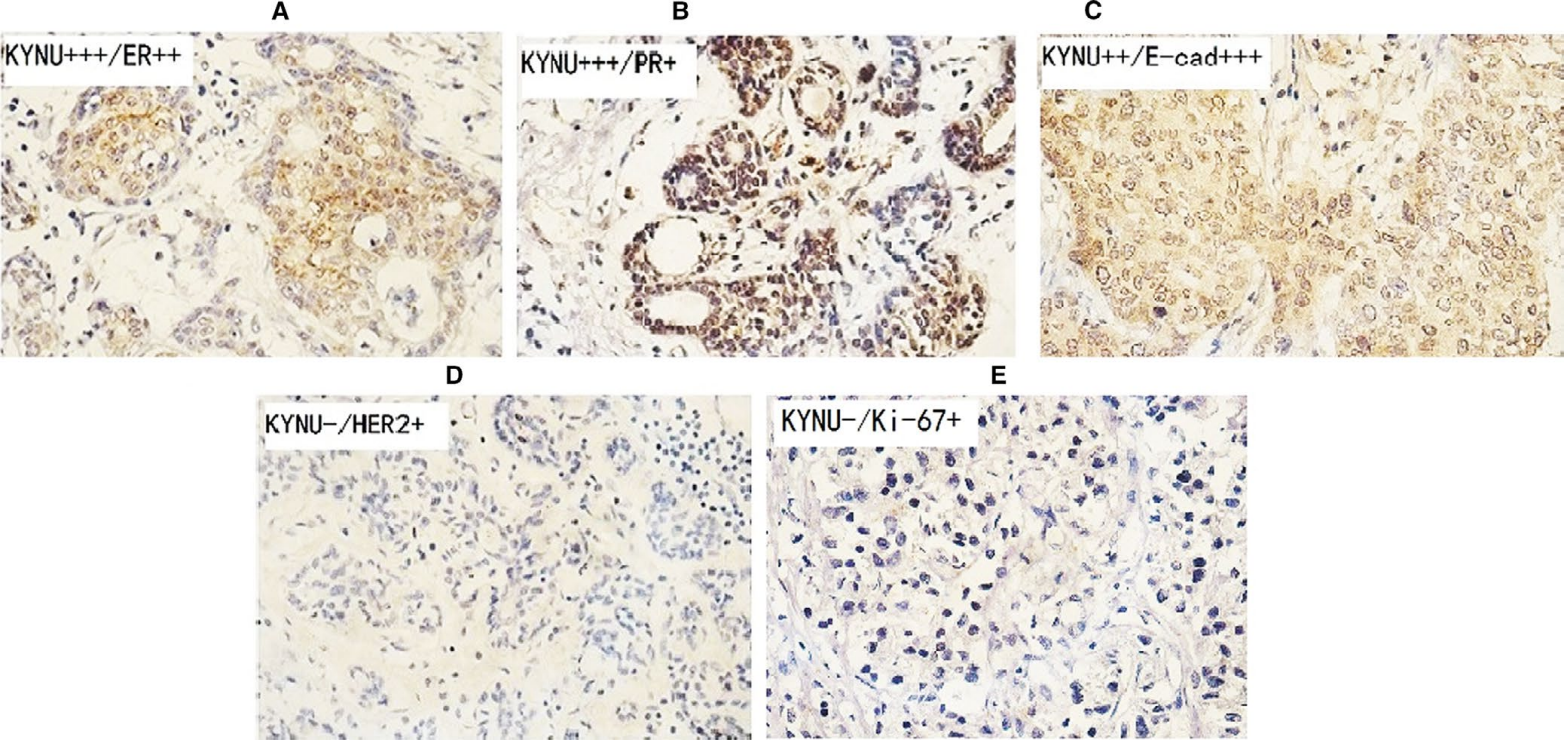
The association of KYNU expression with the biomarkers of BC. A‐C, The IHC staining of high (++ to +++) KYNU in primary BC tissues of ER++ (A), PR+ (B) and E‐cad+++ (C) expressions. D‐E, The IHC staining of low (−) KYNU in primary BC tissues of HER2+ (D), Ki‐67+ (E) expressions. Microscopic image was at 400× magnitude

**Figure 4:**
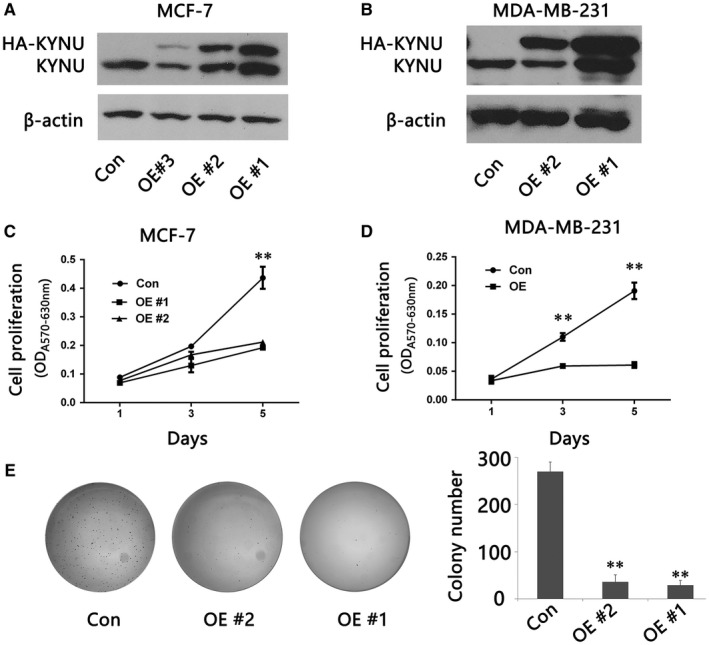
Overexpression of KYNU suppresses BC cell proliferation and tumour development. A, B, Western blot analysis of KYNU expression in control (Con) and different clones of stable KYNU overexpression MCF‐7 (A) or MDA‐MB‐231 cells (B). C, D, MTT assay for cell proliferation of control (Con) and KYNU overexpression (OE) MCF‐7 cells (C) or MDA‐MB‐231 cells (D). E, Soft agar colony formation analysis of control (Con) and KYNU overexpression (OE) MCF‐7 cells. The results in (C), (D) and (E) represent the mean ± SD in triplicate

We further analysed the correlation of KYNU expression with expression of other biomarkers. We found that the expression of KYNU was positively associated with the expression of ER, PR and E‐cadherin (Table 2, Figure 3A‐C), respectively, while negatively related to the expression of HER2 (Table 2, Figure 4D) and Ki‐67 (Table 2, Figure 3E).

### 3.5 Overexpression of KYNU inhibits BC proliferation in culture and colony formation in soft agar

To investigate the role of KYNU in BC development, we established stable KYNU overexpression BC cell lines by transfecting MCF7 and MDA‐MB‐23l cells with KYNU expression plasmid. Several clones with stable KYNU overexpression in each cell type were obtained (Figure 4A,B). MTT assays showed that KYNU overexpression MCF7 or MDA‐MB‐231 cells proliferated significantly slower than control cells (Figure 4C,D). Soft agar colony formation assay showed that KYNU overexpression MCF7 or MDA‐MB‐231 cells had significantly fewer colony formation than control cells (Figure 4E).

### 3.6 KYNU suppresses BC xenograft tumour development in mouse models

**Figure 5:**
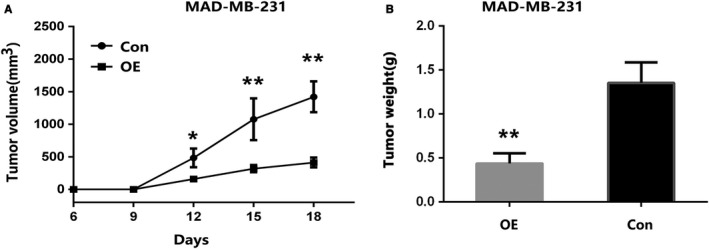
Overexpression of KYNU inhibits BC xenograft tumour development in mouse models. 5 × 10^6^ control vector (Control) or stable KYNU overexpression (KYNU‐OE) MDA‐MB‐231 cells were inoculated s.c. into the flank of six‐week‐old NOD‐SCID female mice. A, B, KYNU‐OE suppressed BC xenograft tumour development in mouse models; tumour development was monitored and measured (A).Tumour weight was significantly higher in control mice than in KYNU‐OE mice (B). (*P < .05, **P < .001)

To further investigate the role of KYNU in BC development, 5 × 10^6^ control vector (Control) or stable KYNU overexpression (KYNU‐OE) MDA‐MB‐231 cells were inoculated subcutaneously (s. c) into the flank of six‐week‐old NOD‐SCID female mice. Tumour development was monitored and measured (Figure 5A). We found that tumour weight was significantly higher in control mice than in KYNU‐OE mice (Figure 5B). MDA‐MB‐231 cells with overexpression of KYNU had significant slower tumour development in mice than control cells (Figure 5A,B), suggesting overexpression of KYNU inhibits BC development.

## 4 DISCUSSION

Breast cancer (BC) is the most common cancer in females and the leading cause of death worldwide,^14^ which are clinically heterogeneous. Because of heterogeneous, clinically BC may be respond poorly to neoadjuvant chemotherapy, or it is respond well to endocrine therapy.^15^ After a loss or reduction of the suppressor genes in its function, normal cells may become into cancer cells with the combination of other genetic changes.^16^ Scientists have learned more about the molecular changes that lead to cancer. The loss of these suppressor genes may be even more important than proto‐oncogene/oncogene activation for the formation of many kinds of human cancer cells.^17^ Therefore, it is important for studying a novel role of some genes in the growth control of breast cancer cells and its relationships with breast cancer. This study showed that changes of KYNU expression in BC primary tissues and BC cell line was involved in a novel role of KYNU in the growth control of breast cancer cells.

Kynureninase (KYNU) located in 2q22.2, which was associated with tryptophan utilization and metabolic diseases including cardiac, renal, and limb defects syndrome 2. However, the role of KYNU in breast cancer (BC) development remains unclear. What do we know about KYNU, it is not reported that KYNU is related to human breast cancer.

Particularly interestingly, among all sections analysed, we found the special expression of KYNU in different cell components of breast cancer tissues and realized that KYNU might have a potential novel function. In BC primary tissues, KYNU protein was highly expressed both in mammary ductal carcinoma cells and in benign myoepithelium cells, but not in lymphocyte and benign mammary gland luminar cells; and the expression of KYNU was very high in well‐differentiated BC cells, and very low in poor‐differentiated BC cells.

Based on the analysis of 137 BC tissues combined with clinical and pathological data, the low expression of KYNU was associated with the malignant characteristics in the primary BC tissues: the expression of KYNU is negatively correlated with clinical BC histological grades and T stages. Kynureninase was highly expressed in well‐differentiated BC (Grade I), while its expression was medium in middle‐differentiated BC (Grade II) and very low or lost in poor‐differentiated BC (Grade III). Similarly, the expression of KYNU was very high in T0 BC and high in T1 BC, while its expression was low in T2 BC and very low in T3 BC.

In this study, the expression of KYNU was significantly related to other biomarkers of BC primary tissues: the expression of KYNU was positively associated with the expression of ER, PR and E‐cadherin respectively; meanwhile, it negatively related to the expression of HER2 and Ki‐67.

Although KYNU has widely been associated with a group of non‐cancer diseases, its role in cancer are unknown, our results suggested that KYNU might play important roles in regulation of BC cell proliferation and differentiation, as overexpression of KYNU suppresses BC cell proliferation and colony formation, and xenograft tumour growth.

To investigate the role of KYNU in BC development, we established stable KYNU overexpression BC cell lines by transfecting MCF7 and MDA‐MB‐23l cells with KYNU expression plasmid. The data showed that KYNU overexpression MCF7 or MDA‐MB‐231 cells proliferated significantly slower than control cells, and KYNU overexpression MCF7 or MDA‐MB‐231 cells had significantly fewer colony formation than control cells. Furthermore, to further investigate the role of KYNU in BC development, stable KYNU overexpression (KYNU‐OE) MDA‐MB‐231 cells were inoculated into NOD‐SCID, and tumour development was significant slower than control cells. The data suggested that overexpression of KYNU inhibited BC development.

Although Song Ping^18^ reported abnormal kynurenine pathway of tryptophan catabolism in cardiovascular diseases, it was not reported that KYNU might be related to human breast cancer. The mammary epithelium is composed of an inner luminal and surrounding myoepithelial cell layer. The presence of cancer cells beyond the myoepithelium defines invasive breast cancer, yet the role of the myoepithelium during invasion remains unclear. A 3D organotypic culture assay to distinguish the functional role of the myoepithelium in regulating invasion and local dissemination.^19^ Time‐lapse microscopy revealed that myoepithelial cells collectively restrain and reinternalize invading Twist1+luminal cells.^19^ Barrier function correlated with myoepithelial abundance and required the expression of α‐smooth muscle actin and P‐cadherin.^19^ The data showed the concept of the myoepithelium as a dynamic barrier to luminal dissemination and implicate both smooth muscle contractility and intercellular adhesion in barrier function.^19^

Here, we report that the expression of KYNU is negatively correlated with clinical BC histological grades and tumour stages. Overexpression of KYNU inhibits BC cell proliferation, colony formation and xenograft tumour development in mouse models.

What do we know, it is not reported that KYNU is related to human breast cancer. Our data showed that KYNU may function as a tumour suppressor in BC. Therefore, KYNU is a promising therapeutic target of BC, but it need take a more further study of KYNU. Here, we only had a try for a Sea horse test (Figure S1). In the future, we will study more about the KYNU molecular changes that lead to breast cancer.

## CONFLICT OF INTERESTS

All authors declare that they have no competing interests.

## AUTHOR CONTRIBUTIONS

Xueping Feng and Yongzhi Xiao planned the study. Yongzhi Xiao and Yingzhe Liu both performed experiments and data analysis. Xueping Feng wrote the study with input from Yongzhi Xiao, Yingzhe Liu, Jinping Lai, Wenjun Yi, Jiu Yang and Tao Du. All authors read and approved the final manuscript.

## ETHICS APPROVAL AND CONSENT TO PARTICIPATE

All procedures performed in studies involving human participants were in accordance with the ethical standards of the Research Ethics committee of the second Xiangya Hospital and the Xiangya Hospital, Central South University, China. This study of BC tissues were obtained after receiving informed consent from all patients, which were used for immunohistochemical (IHC) staining, and certified by the Ethics Committee of the second Xiangya Hospital, Central South University, China. All applicable international, national and/or institutional guidelines for the care and use of animals were followed.

## References

[1] Chen W , Zheng R , Baade PD , et al. Cancer statistics in China, 2015. CA Cancer J Clin. 2016;66(2):115‐132. 26808342 10.3322/caac.21338

[2] Prat A , Pineda E , Adamo B , et al. Clinical implications of the intrinsic molecular subtypes of breast cancer. Breast. 2015;24(Suppl 2):S26‐35. 26253814 10.1016/j.breast.2015.07.008

[3] Fan L , Strasser‐Weippl K , Li JJ , et al. Breast cancer in China. Lancet Oncol. 2014;15:e279‐e289. 24872111 10.1016/S1470-2045(13)70567-9

[4] Koushik SV , Sundararaju B , McGraw RA , Phillips RS . Cloning, sequence, and expression of kynureninase from Pseudomonas fluorescens. Arch Biochem Biophys. 1997;344(2):301‐308. 9264543 10.1006/abbi.1997.0220

[5] Shi H , Enriquez A , Rapadas M , et al. NAD deficiency, congenital malformations, and niacin supplementation. N Engl J Med. 2017;377(6):544‐552. 28792876 10.1056/NEJMoa1616361

[6] Schwarcz R , Bruno JP , Muchowski PJ , Wu H‐Q . Kynurenines in the mammalian brain: when physiology meets pathology. Nat Rev Neurosci. 2012;13(7):465‐477. 22678511 10.1038/nrn3257 PMC3681811

[7] Charlton KG , Johnson TD , Hamed AT , Clarke DE . Cardiovascular actions of kynuramine and 5‐hydroxykynuramine in pithed rats. J Neural Transm. 1983;57(4):199‐211. 6140297 10.1007/BF01248993

[8] Allegri G , Benassi CA , Boccù E , De Nadai A , Persissinotto B . Tryptophan pyrrolase, kynureninase and kynurenine transaminase activities of human renal tumours. Br J Cancer. 1965;19(4):754‐760. 5862656 10.1038/bjc.1965.88 PMC2071386

[9] O'Farrell K , Fagan E , Connor TJ , Harkin A . Inhibition of the kynurenine pathway protects against reactive microglial‐associated reductions in the complexity of primary cortical neurons. Eur J Pharmacol. 2017;5(810):163‐173. 10.1016/j.ejphar.2017.07.008 28688912

[10] Lauvrak SU , Munthe E , Kresse SH , et al. Functional characterisation of osteosarcoma cell lines and identification of mRNAs and miRNAs associated with aggressive cancer phenotypes. Br J Cancer. 2013;109(8):2228‐2236. 24064976 10.1038/bjc.2013.549 PMC3798956

[11] Hammond ME , Hayes DF , Dowsett M , et al. Society of Clinical Oncology/College of American Pathologists guideline recommendations for immunohistochemical testing of estrogen and progesterone receptors in breast cancer (unabridged version). Arch Pathol Lab Med. 2010;134:e48‐e72. 20586616 10.5858/134.7.e48

[12] Wolff AC , Hammond M , Hicks DG , et al. Recommendations for human epidermal growth factor receptor 2 testing in breast cancer: American Society of Clinical Oncology/College of American Pathologists clinical practice guideline update. J Clin Oncol. 2013;31:3997‐4013. 24101045 10.1200/JCO.2013.50.9984

[13] Liu JB , Feng CY , Deng M , et al. E‐cadherin expression phenotypes associated with molecular subtypes in invasive non‐lobular breast cancer: evidence from a retrospective study and meta‐analysis. World J Surg Oncol. 2017;15(1):139. 28764784 10.1186/s12957-017-1210-8 PMC5539617

[14] Liu B , Yi Z , Guan X , Zeng YX , Ma F . The relationship between statins and breast cancer prognosis varies by statin type and exposure time: a meta‐analysis. Breast Cancer Res Treat. 2017;164(1):1‐11. 28432513 10.1007/s10549-017-4246-0

[15] Colleoni M , Munzone E . Navigating the challenges of endocrine treatments in premenopausal women with ER‐positive early breast cancer. Drugs. 2015;75(12):1311‐1321. 26177891 10.1007/s40265-015-0433-7

[16] Dumitrescu RG , Marian C , Krishnan SS , et al. Familial and racial determinants of tumour suppressor genes promoter hypermethylation in breast tissues from healthy women. J Cell Mol Med. 2010;14(6B):1468‐1475. 19799643 10.1111/j.1582-4934.2009.00924.x PMC3829013

[17] Xie G , Chen H , Jia D , et al. The SWI/SNF complex protein Snr1 is a tumor suppressor in drosophila imaginal tissues. Cancer Res. 2017;77(4):862‐873. 27923836 10.1158/0008-5472.CAN-16-0963 PMC7885033

[18] Song P , Ramprasath T , Wang H , Zou MH . Abnormal kynurenine pathway of tryptophan catabolism in cardiovascular diseases. Cell Mol Life Sci. 2017;74(16):2899‐2916. 28314892 10.1007/s00018-017-2504-2 PMC5501999

[19] Sirka OK , Shamir ER , Ewald AJ . Myoepithelial cells are a dynamic barrier to epithelial dissemination. J Cell Biol. 2018;217(10):3368‐3381. 30061105 10.1083/jcb.201802144 PMC6168248

