# Supplementary figures and images for: A novel role of kynureninase in the growth control of breast cancer cells and its relationships with breast cancer
Source: J Cell Mol Med. 2019 Jul 22;23(10):6700–7. doi: 10.1111/jcmm.14547 (PMC6787497; doi:10.1111/jcmm.14547)

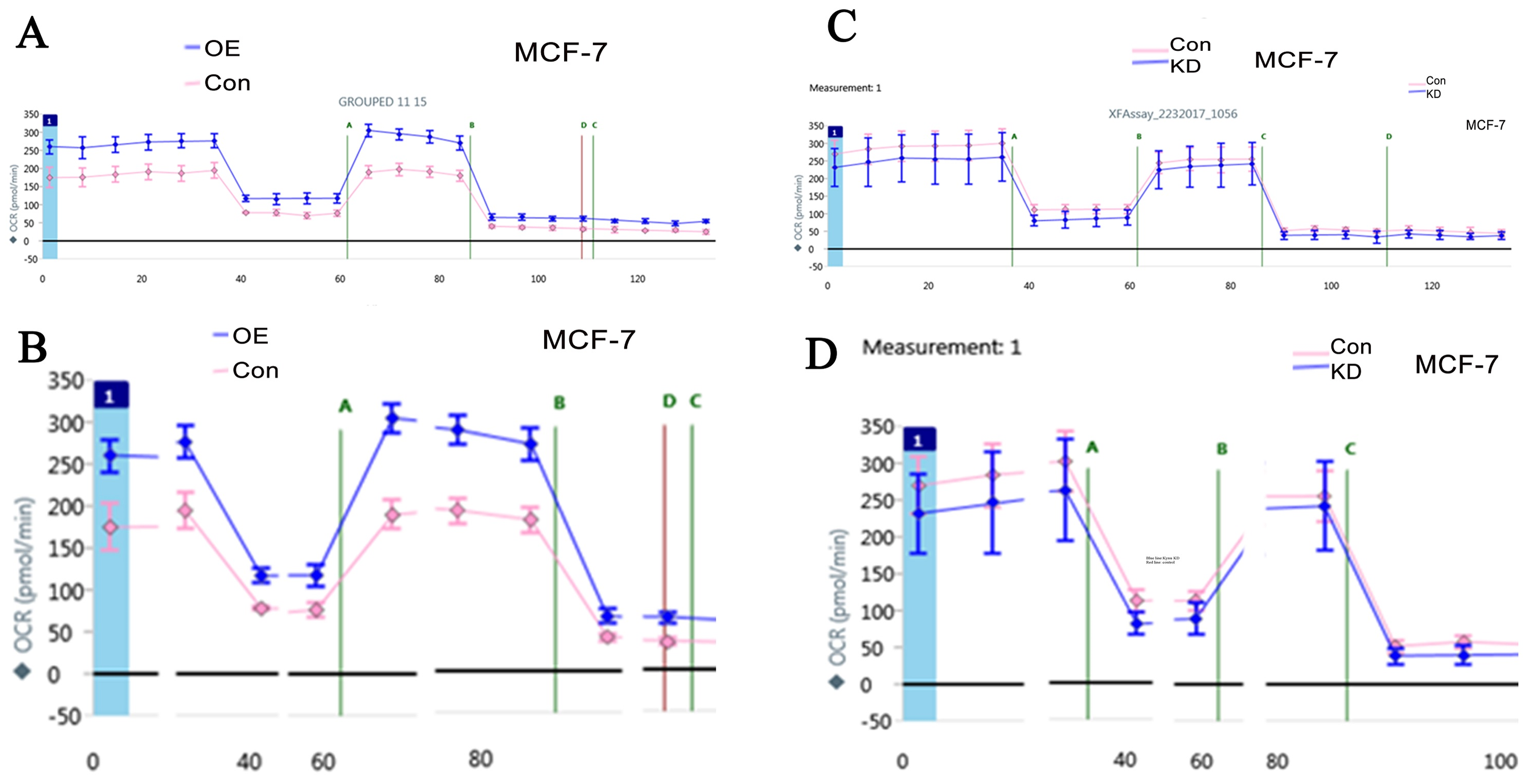

Supplement: Supplementary file 1 [file JCMM-23-6700-s001.tif]
